# Report of Deaths in the City of Buffalo for the Month of May, 1862

**Published:** 1862-07

**Authors:** 


*Report of Deaths in the City of Buffalo for the month of May,* 1862. Accident 6, Accident by drowning 1, Anæmia 1, Angina 1, Apoplexy Cerebral 3, Bronchitis 2, Consumption 17, Convulsions 15, Croup 4, Delirium Tremens 3, Diabetis Millitus 1, Diarrhœa 2, Disease of the Brain 1, Disease of the Heart 5, Disease of the Liver 1, Dropsy general 1, Emphysema 1, Epilepsy 1, Erysipelas 1, Fever Puerperal 1, Fever Scarlet 2, Fever Typhoid 3, Fever Typhus 1, Gangrene 1, Hernia 1, Inflammation of Bowels 2, Inflammation of Brain 7, Inflammation of Brain and Meninges 5, Inflammation of Lungs 18, Inflammation of Lungs Typhoid 3, Intemperance 2, Marasmus 1, Measles 1, Old age 2, Rheumatism 1, Scrofula 1, Tetanus 1, Unknown 2, Whooping Cough 1. Total, 124. Ages—Between 1 and 30 days 6, between 1 and 6 months 13, between 6 months and 1 year 11, between 1 and 3 years 15, between 3 and 5 years 5, between 5 and 10 years 5, between 10 and 20 years 7, between 20 and 30 years 10, between 30 and 40 years 10, between 40 and 50 years 17, between 50 and 60 years 7, between 60 and 70 years 10, between 70 and 80 years 4, over 80 years 1, still born 3, unknown 3. Total, 127. Certified by regular Physicians at the Public Institutions 17; by regular Physicians in the City at large 59; by irregular practitioners 16; by Coroner 9; by Undertakers 26. Total, 127.

